# Future-Oriented Happiness: Its Nature and Role in Consumer Decision-Making for New Products

**DOI:** 10.3389/fpsyg.2020.00929

**Published:** 2020-05-15

**Authors:** Debora Bettiga, Lucio Lamberti

**Affiliations:** Department of Management, Economics and Industrial Engineering, Politecnico di Milano, Milan, Italy

**Keywords:** anticipated happiness, anticipatory happiness, Theory of Planned Behavior (TPB), micro-facial expressions, decision-making process

## Abstract

Cognitive evaluations only partially explain the consumer purchasing patterns, especially when consumers approach a product for the first time. In such an encounter, consumers anticipate the emotions they might experience as a result of their decision, as they cannot realistically evaluate product performances. The work investigates the nature and the influence of these future-oriented emotions, namely anticipated and anticipatory happiness, in the first encounter with new products. Through a first laboratory study, adopting both physiological (micro-facial expressions analysis) and self-reported measures, we confirm the distinction between anticipated and anticipatory happiness. We further show the differential impact of these two emotional constructs on the consumer decision-making process by grounding on the Theory of Planned Behavior (TPB). Through a second study, based on a questionnaire, we further investigate the role of anticipated happiness within the TPB. We show that anticipated happiness is a pervasive emotional construct that influences all stages of the intention formation process. We discuss how these findings enrich existing knowledge on the interplay between cognitive and affective components of the decision-making process for new products. Moreover, we offer a methodological contribution to the use of physiological methods to assess emotions.

## Introduction

The first encounter with a product represents a strong determinant of product adoption (Mugge et al., 2005; Aichner, 2012; Hui et al., 2013). Positive consumer experience is essential in such a touchpoint (Kumar and Garg, 2010; Jun et al., 2019), as consumers cannot realistically evaluate product performances, but only imagining how using that product would be (Palmer and Koenig-lewis, 2014). If research affirms that pure cognitive evaluations only partially explain consumer purchasing patterns (Moons and De Pelsmacker, 2012), the need for considering emotions alongside rationality is even stronger when it comes to consumers approaching products for the first time (Palmer and Koenig-lewis, 2014).

Future-oriented emotions, namely anticipated and anticipatory emotions, have been proposed in the literature as the anticipation of future emotional states that arise when consumers imagine or simulate product consumption (Chang, 2016). For instance, a consumer who envisions or feels that using a particular product will make him/her happy in the future (Baumgartner et al., 2008).

However, the conceptualization of future-oriented emotions and their impact on the purchasing process are largely unexplored. Concerning the first issue, a still fuzzy distinction exists between anticipatory and anticipated emotions, with emotions that have not been precisely classified into one of the two categories (Baumgartner et al., 2008; Carrera et al., 2012). About the second issue, very few studies investigated future-oriented emotions effect on consumer purchasing process (Bettiga and Lamberti, 2017, 2018). Prior research mostly investigates contexts of uncertainty or risk, such as anticipated emotions of gambling or regret (Coricelli et al., 2007) or personal goals, such as losing weight or obtaining a tenure position (Perugini and Bagozzi, 2001). A third shortage of extant research refers to the kind of future-oriented emotions explored. Most researchers focused their attention on negative emotions such as regret, guilt or embarrassment (Kim et al., 2013; Onwezen et al., 2014; Turel, 2016; Londono et al., 2017; Khan et al., 2019), probably due to the evaluation of negative emotions as stronger predictors of behavioral intentions than positive emotions (Baumgartner et al., 2008).

However, recent studies recognize positive emotions, particularly happiness, as relevant drivers of decision-making process (Nicolao et al., 2009; Mogilner et al., 2011; Bhattacharjee and Mogilner, 2014; Petersen et al., 2018; Cloarec et al., 2019). This opens new space for investigation of positive emotions for which boundaries, as well as effects on the purchase process, are still unclear (De Keyser and Lariviere, 2014; Ayadi et al., 2017).

In light of these considerations, this work aims, through two exploratory studies, at deepening the understanding of the nature and the influence of future-oriented happiness in the first encounter with a new product. Such relationships are studied through both self-reported (a questionnaire) and physiological (Facial Action Coding System) methods. By doing that, the works wants to contribute to extant literature by shedding light on the still vague conceptualization of anticipated and anticipatory happiness, supporting their conceptual distinction. Furthermore, it aims to explore their effect on the consumer purchasing patterns, by extending the Theory of Planned Behavior (TPB) and enriching existing knowledge on the interplay between cognitive and affective components of the decision-making process. Finally, the study introduces the use of physiological methods to assess emotions, thus offering a methodological contribution.

## Anticipated and Anticipatory Emotions in Decision-Making

Academic research displays a raising interest toward irrational and affective dimensions of consumer decision making, with many attempts to improve cognitive models of decision-making by including emotional constructs. Emotions spontaneously affect the decision-making process (Moons and De Pelsmacker, 2012). They may constitute pivotal mediating variables in behavioral responses (Schoefer and Diamantopoulos, 2008) or direct drivers of the purchase decisions (Di Muro and Murray, 2012). This is particularly true when it comes to consumers approaching products or services for the first time (Palmer and Koenig-lewis, 2014; Bettiga et al., 2017). Indeed, consumers tend to evaluate new stimuli through the feelings they may elicit (Holbrook and Hirschman, 1982; George et al., 2016). Individuals, indeed, are not able to realistically evaluate product usage in the pre-consumption stage, thus they tend to rely on emotions in such decision-making process (Palmer and Koenig-lewis, 2014).

Among cognitive theories adopted to analyze the decision-making process, the TPB is probably the most recognized and adopted model (Ajzen, 1991). Its key concepts and relationships have been proved effective in predicting consumer intentions for a wide variety of products and behavior (Pavlou and Fygenson, 2006; Chen and Tung, 2014; Hassan et al., 2016). TPB has constituted the core theory on which to develop newer models to explain purposive behaviors (Perugini and Bagozzi, 2001; Londono et al., 2017). Comparatively to the Theory of Reasoned Action (TRA- Fishbein and Ajzen, 1975), from which it originates, it includes an additional major construct, the Perceived Behavioral Control (PBC). PBC showed to improve the prediction of behavioral intention and behavior, especially when the behavior is not completely under volitional control (Madden et al., 1992). According to the TPB, behavioral intention (I), a predictor of behavior, is influenced by attitude (A) – the overall evaluation one performs of certain behavior and its possible consequences -, subjective norms (SNs) – the perceived social pressure – and PBC – the perceived ease or difficulty associated with a specific behavior.

The TPB is a widely applied theory to understand and predict human behavior (Pavlou and Fygenson, 2006; Chen and Tung, 2014; Hassan et al., 2016). However, many authors argue that its efficacy could be improved by overcoming present limitations. The first limitation regards the intention-behavior gap where, theoretically, “behavioral intentions are motivational factors that capture how hard people are willing to try to perform a behavior” (Pavlou and Fygenson, 2006, p. 117). However, the intention may not be a good predictor of the final behavior as people may fail to act on their stated intentions. The causes of this gap can sometimes be found in the methodology adopted to measure both intention and behavior (Hassan et al., 2016). The second limitation of the TPB concerns the inability of the model to predict behaviors that are not driven by intentions such as habits and impulsive buying (Wood and Neal, 2009; Verplanken and Sato, 2011). However, even for deliberate behaviors, the TPB may fail in capturing all determinants of such intentions. Indeed, the TPB is highly cognitive and relies on the assumption that most behaviors people engage in are rational and under their control. Thus, it tends to perform less efficiently for behaviors grounded on strong irrational and affective elements (Kim et al., 2013). For these reasons, various studies tried to improve the predictive power of TPB by including emotional constructs (Kim et al., 2013; Onwezen et al., 2014; Turel, 2016; Londono et al., 2017). Following prior studies, we rely on the TPB as the building block of on which to develop our model.

Emotions are the result of the appraisal of a specific situation, they are highly subjective and they depend on the person that performs this evaluation. They “have a specific referent,” meaning that they arise only as a response of an “appraisals one makes for something of relevance to one’s well-being” (Bagozzi et al., 1999, p. 185). The potential consequences of the current decision heavily influence consumers’ decision making too (Bagozzi et al., 2016). Consumer anticipation, indeed, is as a mental process by which consumers envision the impact that a certain consumption decision may have on the self in the future (Vichiengior et al., 2019). Hence, when consumers imagine or simulate product consumption, they generate affective expectations about how using that product could make them feel (Chang, 2016), the so-called anticipated and/or anticipatory emotions.

Baumgartner et al. (2008) proposed a distinction between Anticipatory and Anticipated Emotions, confirmed in further studies (Carrera et al., 2012; Barsics et al., 2016; Xu and Guo, 2019). Anticipatory Emotions (A_Y_Es) are affective reactions that are experienced in the present “due to the prospect of a desirable or undesirable future event” (Baumgartner et al., 2008, p. 685). They consist of current and real affective responses to future events. Anticipated Emotions (A_D_Es), instead, arise when a person project to experience certain emotions in the future, in response to an event. Even though they could be accompanied by vivid visualizations at the present, they represent predictions of future emotional states. Nevertheless, these forecasted emotions can shape present consumer behavior through a self-regulatory function.

Anticipated Emotions rely on “cognitive-evaluative dimensions” while Anticipatory Emotions are connected to “experiential-current reactions” (Carrera et al., 2012, p. 274).

Both Anticipated and Anticipatory Emotions impact behavioral intentions, where A_D_Es have stronger motivational effects than A_Y_Es. Moreover, negative emotions are stronger predictors of behavioral intentions than positive emotions (Baumgartner et al., 2008). Anticipated Negative Emotions, which include regret, shame, sadness, embarrassment, have been proved to influence cognitive evaluation, being direct predictors of behavioral intention in the TPB framework (Kim et al., 2013; Londono et al., 2017). This suggests that decision-making models such as the TPB could largely benefit by accounting emotions in intentions formation. Anticipated pride and guilt shown to impact the TPB by partially mediating the impact of attitude and subjective norms on intention (Onwezen et al., 2014). Among Anticipated Positive Emotions, happiness and hope proved to induce a more favorable attitude toward the product (Chang, 2016). Besides, the occurrence likelihood of the target future event influences the experience of A_Y_Es, but it is not related to A_D_Es. This work investigates such issues in two exploratory studies. Specifically, Study 1 examines the nature and the impact of anticipated emotions and anticipatory emotions along the decision-making process. Study 2 deepens the role of anticipated happiness on individuals’ decisional patterns by analyzing its role in the TPB.

## Study 1

### Research Questions Development

Research acknowledges the importance of emotions in relation to consumer behavior (Argo et al., 2006; Soscia, 2007; Baumgartner et al., 2008; Schoefer and Diamantopoulos, 2008; Nicolao et al., 2009; Mogilner et al., 2011; Di Muro and Murray, 2012; Kim et al., 2013; Bhattacharjee and Mogilner, 2014; Onwezen et al., 2014; Sun et al., 2015; Chang, 2016; Turel, 2016; Bettiga et al., 2017; Londono et al., 2017). Despite that, a lack of knowledge is evident on the impact of positive emotions in the decision-making processes. Researchers mainly focused their attention on negative emotions such as regret, guilt, embarrassment (Kim et al., 2013; Onwezen et al., 2014; Pletti et al., 2016; Turel, 2016; Londono et al., 2017), probably because negative emotions have been recognized as stronger predictors of behavioral intentions than positive ones (Baumgartner et al., 2008). Few studies focused on positive emotions, even if they recognize them, particularly happiness, as a relevant driver of the decision-making process (Mogilner et al., 2011; Martínez-Ruiz et al., 2017).

Happiness, defined as “a state of well-being and contentment; a pleasurable or satisfying experience” (Mogilner et al., 2011, p. 430) revealed a strong positive relationship with consumption (Mogilner et al., 2011; Wang et al., 2019) making this construct particularly relevant in the study of consumer consumption processes. However, most of the studies to date focused only on the experiential and indulgent sides of purchase. Research studied happiness in relation to material vs. experiential purchases (Nicolao et al., 2009), investigate whether happiness could vary across ordinary and extraordinary experiences (Bhattacharjee and Mogilner, 2014) and analyzed the relationship between indulgent purchases and happiness (Petersen et al., 2018). The current research moves in a novel direction, by exploring happiness in the first encounter with new products, to uncover insights on this powerful but still little explored emotion. Finally, prior studies do not clearly distinguish between anticipated and anticipatory emotions and their respective impact on consumer decision-making (Baumgartner et al., 2008; Carrera et al., 2012). So, the first objective of this work is to investigate if Anticipated and Anticipatory Emotions are indeed two different constructs. More formally:

***RQ1:*** *Anticipated and Anticipatory Happiness are two distinct emotional reactions.*

The second objective of the study is to investigate if Anticipatory and Anticipated happiness do have an impact on the decision-making process. To do that, we will explore the impact of Anticipated and Anticipatory Happiness on the three acknowledged predictors of intention (A, SNs, and PBC) in the Theory of Planned Behavior. Thus:

***RQ2:*** *Anticipated and Anticipatory Happiness have a relationship with Attitude, Subjective Norms and Perceived Behavioral Control.*

### Materials and Methods

The study employs two different methodologies to assess Anticipated and Anticipatory Happiness elicited by the same stimulus. The former is evaluated through self-reported methods, by utilizing a questionnaire, while the latter is measured through autonomic physiological responses, by employing a microfacial expression reader. The choice of these two methodologies is due to the nature of Anticipated and Anticipatory Emotions. Anticipated Emotions require the individual to imagine a future consumption situation and try to envision how they would feel in that context. So, since they require a cognitive elaboration, this effort could be better captured by employing a self-reported method like a questionnaire. Anticipatory Emotions, instead, are emotional responses experienced in the present moment when one thinks about a future event, hence they are better evaluated through the affective physiological responses, the spontaneous and unconscious reactions to the stimulus. In this regard, we use the Facial Action Coding System (FACS), firstly developed by Paul Ekman and Wallace V. Friesen in 1978 and largely used in marketing researches to assess emotional responses (Teixeira et al., 2012; Hernández-Fernández et al., 2019). It can determine the emotional state of the interviewed subject through the analysis of the micro-expressions (fear, anger, surprise, disgust, happiness, and sadness), which are involuntary contractions of the facial muscle.

#### Stimuli Selection

The chosen stimuli were two teaser videos advertising new products, which lasted ∼30 s each. The use of two different videos guarantees the capability to accurately assess emotion intensity. Moreover, their length is recognized to be appropriate to elicit emotional responses in the subject (Ménard et al., 2015). The advertised products belonged to two different categories: a personal care product (a teeth whitening pen) and an electronic good (a laser keyboard). When choosing the products, two main constraints were set: the product had to be relatively new to the respondent to avoid being influenced by previous experiences of use. When past experiences are available, people tend to rely more on those experiences and engage less in processing information (Palmer and Koenig-lewis, 2014). This results in a weaker relationship between A, SNs, PBC and intention to purchase the product (Kidwell and Jewell, 2008). Similarly, also the brand name was left out on purpose since it works as a memory cue which pushes consumers to retrieve past information to guide their future decisions.

#### Experimental Design and Sample

A laboratory experiment was conducted, involving 50 Italian participants. Such sample size is more than satisfactory for physiological studies, usually grounded on <20 subjects (Stoll et al., 2008; Santos et al., 2012; Teixeira et al., 2012; Hernández-Fernández et al., 2019). The experiment lasted 10 min and it was carried inside a university laboratory. Participants selected did not have to carry cardiac diseases, acute visual impairments and they should not have participated in neuromarketing experiments in the previous 6 months. Subjects were invited to sign an authorization module which informed them about the purpose of the experiment, the technical equipment used to perform the experiment, the possibility to interrupt the experiment whenever they wanted and the data treatment. Then, they were invited to sit in front of a monitor, where a webcam was installed in front of them to record their expressions while watching the two product advertising videos and answering a questionnaire. The subjects were recommended to avoid touching or obscuring their face while watching the videos and to remove their hair from their face (a fringe, for example, could impede the clear recognition of the micro facial expression in the analysis phase). At the beginning of the first video, a neutral image was displayed on the screen for 20 s, to collect the basic expression of the subject (i.e., the expression the subject has when no external stimuli are provided).

#### Questionnaire Development

The questionnaire was mainly developed grounding on Ajzen (2006). It consisted of three main sections: the first aimed at gathering descriptive information about the respondents such as gender, age, level of education, type of job and nationality. The other two sections were needed to collect data about the original TPB factors (A, SNs, PBC, and I) and the extended factor (A_D_H) for the two different products. At the beginning of each section, a brief description of the product was presented, followed by the teaser advertising video. Questions were introduced with a short instruction about how to answer them.

The questions testing the Anticipated Happiness’ construct were assessed using 7-points Likert scales ranging from “not at all” to “extremely.” Anticipated Happiness was assessed with four items (Laros and Steenkamp, 2005; Mogilner et al., 2011; Chang, 2016), asking participants to envision how they would feel about using the product in the future and the projected impact of the products on their lives. Specifically, we asked them to rate how pleased would they feel about using the product, if the idea of using it would make them feel better and if the product could solve annoying problems and improve their everyday life. The questions testing Attitude, Subjective Norms, Perceived Behavioral Control, and Intentions were assessed using 7-points Likert scales ranging from “strongly disagree” to “strongly agree.” Attitude toward the products was assessed through five items (Ajzen, 2006). Participants had to rate how much they thought the product could be “a good idea,” “pleasant,” “valuable,” “enjoyable,” and “beneficial” for them. Subjective Norms were measures through three items (Ajzen, 2006): participants had to indicate whether their relevant others (family and friends) would agree with their purchase and then they had to rate their propensity to listen to the advice of their relevant others. Perceived Behavioral Control was measured with five items (Ajzen, 2006; Hsu et al., 2017) able to capture the perceived ability to use the product and the extent to which respondents had the resources needed to use it. The intention to buy the product was measured using three items (Ajzen, 2006) that evaluated the willingness of the respondents to acquire the product. The questionnaire was translated into Italian, the mother tongue of the participants. A preliminary test of the questionnaire was carried out on 10 subjects to gather relevant feedbacks regarding distinct aspects like syntax, easiness of comprehension and overall clarity.

#### Microfacial Expressions Assessment

Participants were recorded while watching the teaser videos. Each video was then analyzed using *Noldus Face Reader, version 7.* The software automatically assesses the intensity of a series of six emotional states of the subject: happiness, fear, anger, disgust, surprise, and sadness. The output is a temporal track where all the changes in the subject’s expressions are analyzed and classified into emotions (changes in the subject’s face are recorded every 66 ms). The emotion intensity ranks from 0 to 1 and values below 0.05 are considered background noise.

### Results

#### Preliminary Analysis

Two subjects for the personal care product stimulus and five for the electronic device were not considered for data analysis due to low quality of the facial expression recording (mainly subjects partially covering their faces with hands during the experiment). Once the cleaning session of the data had been performed, the emotional track of each participant was calibrated. The calibration phase is needed to remove person-specific biases, such as a facial expression naturally skewed toward anger. For each participant, it was necessary to select 2 s of video recording to be used as an input for the calibration analysis. This time interval was taken while the subject was staring at the neutral image at the beginning of the first video. If for some reason the expression of the subject was not considered neutral, the calibration was done considering another part of the track where the subject was as neutral as possible (e.g., when answering to the demographic questions in the questionnaire).

#### Descriptive Analysis

The sample was composed of 40% men and 60% women. Age distribution was the following: 2% of the respondents aged between 19 and 25 years old, 20% between 26 and 35, 32% between 36 and 45 and the remaining 46% aged over 45 years old. Regarding the education level, 70% of the participants attended high school, 28% got a degree (Bachelor or Master degree) and 2% obtained a Ph.D. Analysing the type of job, the majority of the respondents (66%) worked as employees, 20% were entrepreneurs, 2% declared to be retired and the remaining 12% was unemployed (no students were present in the sample). All the respondents were Italian. With one exception, all respondents never used before the advertised products and this is aligned with our purpose to avoid past experiences that influence the evaluation of the products. Table 1 shows the descriptive statistics of the TPB model constructs evaluated, divided by product. Table 2 provides the descriptive statistics of the six basic emotions that were detected using the face reader. The intensity of the emotions detected in the laboratory environment resulted quite low as respondents mainly display a neutral expression during the session (mostly, the intensity of the emotions recorded scored values lower than 0.05/1, not considered manifestations of emotion but a background noise). For both stimuli more than half of the participants did not manifest any emotion.

**TABLE 1:** Descriptive statistics of A, SNs, PBC, and A_D_H.

	Electronic device		Personal care product	
Construct	*M*	*SD*	*M*	*SD*
A*	5.03	1.53	5.37	1.93
SNs**	21.51	13.25	26.02	14.51
PBC*	6.18	0.78	6.14	0.90
A_D_H*	4.81	1.44	5.28	1.78

*Max value 7; **Max value 49.

**TABLE 2:** Descriptive statistics of Anticipatory Emotions.

	Electronic device		Personal care product	
Anticipatory emotions*	*M*	*SD*	*M*	*SD*
Happy	0.029	0.047	0.043	0.078
Sad	0.083	0.108	0.105	0.134
Angry	0.044	0.063	0.053	0.070
Surprised	0.014	0.034	0.027	0.055
Scared	0.005	0.018	0.010	0.026
Disgusted	0.033	0.057	0.037	0.075

*Values between 0 and 1.

#### RQ1 Results

**FIGURE 1:**
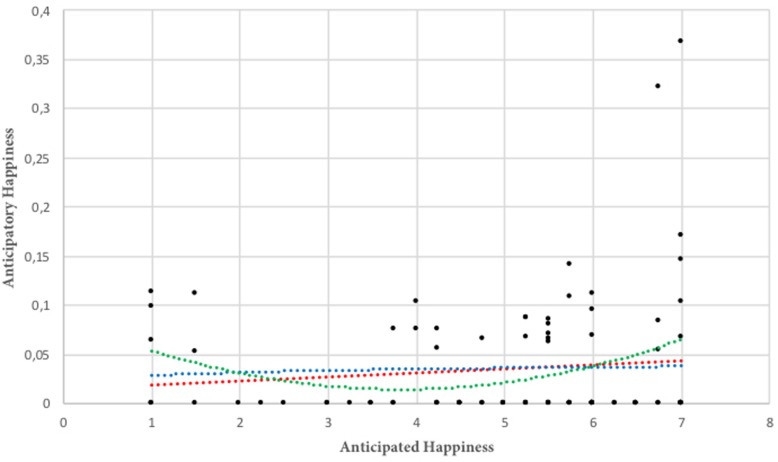
Relationship between anticipated and anticipatory happiness.

The first part of the analysis investigated whether there is a relationship between Anticipated and Anticipatory Happiness. To the best of our knowledge, there is no previous evidence that supports a specific type of association between them. Thus, three possible regression models were investigated: linear, logarithmic and polynomial. Figure 1 plots the regression lines for each tested relation: the red dotted line represents the linear regression; the blue and the green represent the logarithmic and the polynomial regression lines respectively. Results confirms our expectations, demonstrating that the two variables are not related (*R*_lin_^2^ = 0.011; *R*_log_^2^ = 0.002; *R*_polinomial_^2^ = 0.070; number of observations = 93).

#### RQ2 Results

Respondents were divided into two clusters based on the fact that they manifested the emotions of Anticipatory Happiness through their facial expression or not, at least in response to one stimulus. Following, one-way analyses of variance (Single Factor ANOVA) were run to understand if there was any difference in the intention predictors among the two clusters. Results (Table 3) revealed that subjects with higher (lower) tendency to show A_Y_H through facial expressions declared lower (higher) levels of PBC. Thus, more “emotional” subjects perceived on average to have less control. Conversely, high-control individuals were those who did not express A_Y_H through facial expressions during any of the two stimuli. Further, Anticipated Happiness was analyzed in relationship with intention antecedents. Given that these variables (A, SNs, PBC, and A_D_H) were assessed with the same measurement method (questionnaire), a regression analysis seems appropriate to assess relationships between A_D_H and each of the three predictors. Results show that A_D_H could qualify as a predictor of all constructs of A, SNs, and PBC. A first regression was calculated to predict A based on A_D_H. A significant regression equation was found [*F*(1, 91) = 771.45; *p* < 0.000] with an R^2^ of 0.89. A second regression analysis calculates SNs based on A_D_H, with results of [*F*(1, 91) = 69.69; *p* < 0.000] and an R^2^ of 0.43. A third regression predicts PBC based on A_D_H, with the following results: [F(1, 91) = 10.29; *p* < 0.002] with an R^2^ of 0.10. Looking at the R^2^ values it can be noticed that the stronger relationship is between A_D_H and A, with an explained variance of 89%, while A_D_H explains around 43% of the variance in SNs and 10% of the variance in PBC.

**TABLE 3:** One-way analysis of variance and means for PBC by AyH.

Measure	Mean		*F*(1,91)	*p*-value
	AyH			
	Yes	No		
PBC	5.93	6.33	5.303	0.024

### Discussion

This study aimed to understand whether Anticipated and Anticipatory Happiness constitute two different emotional reactions and their role in the decision-making process. The choice to investigate consumer happiness is driven by two main reasons. First, the fact that this emotion has been proven to play a crucial role in shaping consumer behavior (Nicolao et al., 2009; Bhattacharjee and Mogilner, 2014; De Keyser and Lariviere, 2014; Ayadi et al., 2017; Petersen et al., 2018). Second, the intent to expand prior literature on consumer emotions, which has traditionally focused on the role of negative emotional responses (Onwezen et al., 2014; Turel, 2016; Londono et al., 2017).

To test our first research question, two methods were employed, to account for previously proposed differences between the two categories of emotions (Baumgartner et al., 2008). Results provide support for H1, confirming that there is a distinction between Anticipated and Anticipatory Happiness, supporting Baumgartner et al. (2008) arguments of divergence between the constructs. Even more remarkably, we contribute methodologically by measuring anticipatory emotions (more consistently to their definition) through autonomic, objective measures, and demonstrating the divergence between them and self-reported anticipated emotions. This is consistent with previous studies showing how biofeedback and self-reported scales tend to diverge as they tackle with different “mechanisms” of the human brain (e.g., Bettiga et al., 2017). The implications of these outcomes open interesting developments in conceptual terms, like the study of the interaction between A_D_Es and A_Y_Es, and also in methodological terms, suggesting how biofeedback may provide a richer understanding of product adoption processes.

Furthermore, study 1 frames A_D_Es and A_Y_Es in the TPB. Results suggest that whereas higher levels of anticipatory emotions are reached, decreasing levels of self-reported PBC are observed. We find this result intriguing and somehow controversial. We interpret this phenomenon contending that A_Y_Es are somehow expressions of an individual’s emotionality, where higher (lower) levels of perceived control may be associated with a weaker (stronger) tendency to show emotions and vice versa. Another possible explanation could be that control perception may inhibit the emotional reactions one has in terms of facial expressions. These two explanations find support in the literature (Biehl et al., 1997; Rhodes et al., 2005; Anggraini and Siswanto, 2016; Hofmann et al., 2019) and are suggested to be further studied in the future.

As per Anticipated Happiness, our results show that A_D_H is arguably a possible antecedent of all intention antecedents (A, SNs, and PBC), even though the strongest impact emerged with attitude. Nonetheless, such an outcome remains unclear and raises the opportunity and the relevance of deepening the relationship between anticipated emotions (and, namely A_Y_H) and the TPB constructs, that is the object of our Study 2. Finally, the results of Study 1 appear consistent with the idea that A_D_H, inherently more cognitive, is mostly linked with the cognitive construct of attitude. Indeed, they both require the subject a mental elaboration effort (attitude requires to evaluate the product while Anticipated Happiness requires to imagine future consumption and understand one’s feeling in that situation). Conversely, A_Y_H, which is less cognitive and more visceral, is mostly linked to the perception of control, which is a highly subjective construct, more related to subjects’ perceptions than to rational assessments. To conclude, findings of Study 1 leave space for further developments on how A_D_E, and more in detail A_D_H, can be an important predictor in the decision-making process.

## Study 2

### Research Questions Development

Findings of Study 1 suggest to deeper investigate the role of A_D_Es and in particular A_D_H in the decision-making process, to improve the capability to explain the intention to purchase new products. Hence, we develop a second study to further explore the role of Anticipated Happiness in the TPB model of decision-making. Grounding on the findings of the first study and on prior studies that propose both empirical and theoretical evidence for supposing that Anticipated Happiness could qualify as an important additional component in the TPB framework, we formulated the following research question:

**RQ3:** *Anticipated Happiness is a direct antecedent of Intention in the Theory of Planned Behavior.*
**RQ4:** *Anticipated Happiness is a direct antecedent of Attitude (a), Subjective Norms (b), and Perceived Behavioral Control (c) in the Theory of Planned Behavior.*
**RQ5:** *Anticipated Happiness partially mediates the impact of Attitude (a), Subjective Norms (b) and Perceived Behavioral Control (c) on Intention in the Theory of Planned Behavior.*

### Methodology

The self-reported method chosen to assess Anticipated Happiness in the first study is also employed in this second one. Thus, the previously stated research questions have been tested using an online questionnaire, with the same measurement scales employed in study 1. The questionnaire has been distributed online on social platforms. The sample of participants is a non-student sample, for which descriptive information have been reported in paragraph Preliminary and Descriptive Analysis. This method allows reaching a larger sample size which is adequate for the objective of testing structural relationships among constructs through Structural Equation Modeling (Henseler et al., 2009).

### Results

#### Preliminary and Descriptive Analysis

The total amount of respondents was 316. Ten subjects were not considered in the analysis due to potential disengaged answering mode (e.g., some records scored the same in all items). The sample was composed of 40% of men and 60% of women. Most of the respondents (74%) were equally split between two age ranges: 19–25 and over 45. Regarding the education level, 3% of the sample did a Ph.D. program, 48% own a degree, 43% did the high school and 6% the middle school. Analysing the type of job, a great part of the population (50%) work as an employee, 29% declared to be students, 13% entrepreneurs, 3% retired and the remaining 5% unemployed. Most of the respondents (90%) were Italian. The majority of the respondents didn’t have previous experience with both the personal care product (only 6% of respondents already used this product) and the electronic device (2% of respondents already used this product). Tables 4, 5 show the main descriptive statistics and the correlation coefficients of the variables postulated in the extended TPB model divided by product.

**TABLE 4:** Descriptive statistics of the extended TPB model – Personal care product.

Construct	*M*	*SD*					
A*	3.48	1.61	1.00				
SNs**	15.02	10.73	0.64	1.00			
PBC*	5.54	1.17	0.18	0.20	1.00		
A_D_H*	3.30	1.49	0.87	0.61	0.13	1.00	
I*	2.87	1.71	0.84	0.56	0.10	0.81	1.00

*Max value 7; **Max value 49.

**TABLE 5:** Descriptive statistics of the extended TPB model – Electronic device.

Construct	*M*	*SD*					
A*	4.00	1.67	1.00				
SNs**	18.20	12.28	0.66	1.00			
PBC*	5.36	1.30	0.39	0.33	1.00		
A_D_H*	3.65	1.64	0.86	0.62	0.28	1.00	
I*	2.90	1.66	0.77	0.61	0.27	0.74	1.00

*Max value 7; **Max value 49.

#### Inferential Statistics

Data were analyzed with a series of Single Factor ANOVA tests to assess whether different groups of respondents were statistically different concerning a specific variable (A, SNs, PBC, A_D_H, and I). Two groups’ discriminants were defined: sex (male/female) and age (the first group included all respondents <25 years old, the second between 26 and 45 years old and the third over 45 years old). When comparing the three age groups, ANOVA was supplemented by the Tukey-Kramer *post-hoc* test, to understand where the differences lied. Tables 6, 7 report the results, respectively, for the health care product and the electronic device. Concerning the personal care product, no significant differences were found between males and females. Conversely, attitude and subjective norms were found to be significantly higher between individuals below 25 years-old and individuals above 45 years-old. Likewise, for the electronic device, ANOVA tests evidence no significant difference between males and females. However, A_D_H, A and SNs were found to be significantly greater for individuals below 25 years-old and individuals above 45 years-old.

**TABLE 6:** One-way analysis of variance and means for personal care product by age.

Measure	Mean			*F*(2,303)	*p*-value
	Age				
	<25	26–45	>45		
A	3.742	3.484	3.211	3.18	0.043
SNs	16.558	15.459	13.071	3.19	0.043

**TABLE 7:** One-way analysis of variance and means for electronic device by age.

Measure	Mean			*F*(2, 303)	*p*-value
	Age				
	<25	26–45	>45		
A	4.295	3.819	3.813	3.059	0.048
SNs	20.944	17.959	15.417	6.088	0.003
A_D_H	3.971	3.497	3.415	3.819	0.023

#### Measurement Model

The data were analyzed with PLS-SEM analysis carried out using *SmartPLS 3.2.7.* We opted for PLS-SEM being an appropriate and robust method to analyze composite models (Hwang et al., 2010; Wong, 2013). Among second- generation techniques, we opted for PLS-SEM, instead of covariance-based SEM, due to the explorative nature of the research and the complexity of the structural model – i.e., several constructs and indicators- (Hwang et al., 2010; Avkiran, 2018), that make the use of PLS-SEM more suitable (Hair et al., 2011). As suggested by Henseler et al. (2009) to achieve robust PLS estimations, the sample size should be at least 10 times the largest number of structural paths pointed to a specific construct in the inner path model. The largest number of structural paths directed to a construct in our inner model is equal to 4 making our sample size adequate.

Before assessing the causal relationships among the constructs of the proposed framework, data were analyzed through a CFA (Confirmatory Factor Analysis) to ensure both the reliability and validity of the reflective measurement model. Indicators’ reliability was tested by looking at the outer loadings: acceptable values have to be above 0.6–0.7 for each measurement item (Hulland, 1999). After this check, only one indicator was dropped since its value was found to be below 0.6. This result supports the theoretical assignment of the indicators to each construct, hypothesized during the questionnaire development.

Internal consistency reliability was evaluated through CR (Composite Reliability) measures, instead of Cronbach’s alphas (Henseler et al., 2009). PLS, indeed, priorities indicators based on their reliability, achieving a more consistent composite than Cronbach’s Alfa, which instead assumes that all indicators are equivalently reliable. All CR values exceed 0.7 for each latent variable, so they are considered acceptable (Henseler et al., 2009). Convergent validity was assessed by looking at the AVE (Average Variance Extracted), which had to be =0.5 for each latent variable (Bagozzi and Yi, 1988), meaning that each latent variable can explain on average more than half of the variance of its block indicators. All constructs had an AVE value greater than the threshold. Discriminant validity has been tested with two approaches. Firstly, the Fornell-Larcker criterion (Fornell and Larcker, 1981), imposing the square root of AVE of each latent variable to be higher than the correlations with all other latent variables. In this way each latent variable has to share more variance with its own set of indicators than with another latent variable representing a different set of indicators. Secondly, it has been tested through the heterotrait-monotrait ratio of correlations (HTMT) based on the multitrait-multimethod matrix (Henseler et al., 2015). Tables 8, 9 present a summary of the results of constructs reliability and validity.

**TABLE 8:** Internal consistency reliability and validity results.

Construct	CR	AVE	A	A_D_H	I	PBC	SNs
A	0.953	0.801	**0.895**				
A_D_H	0.938	0.790	0.868	**0.889**			
I	0.961	0.891	0.805	0.768	**0.944**		
PBC	0.851	0.592	0.300	0.229	0.200	**0.769**	
SNs	0.952	0.869	0.655	0.621	0.583	0.295	**0.932**

*The bold values represent the square root of AVE.*

**TABLE 9:** Heterotrait-Monotrait Ratio (HTMT) results.

Construct	A	A_D_H	I	PBC
A_D_H	0.938			
I	0.848	0.824		
PBC	0.342	0.263	0.226	
SNs	0.703	0.677	0.621	0.339

#### Structural Model

Once the suitability of the outer model was tested, it was necessary to assess the quality of the inner model and to check whether the posited relationships among the constructs were supported by the data. A PLS algorithm with 300 iterations was run to obtain the coefficients of the structural path and the variance of the dependent variable(s) explained by the independent variables (R^2^ and R^2^_adjusted_). Then the significance of the path coefficients was tested using the bootstrap approach with 5,000 re-samples (Londono et al., 2017).

Table 10 shows that all the propositions are supported. Therefore, A_D_H can play a role in the TPB both as a direct antecedent of all original constructs (A, SNs, PBC, and I) and as a partial mediator of the existing relationships among the constructs. These results highlight the pervasive role of anticipated positive emotions in intention formation, suggesting that A_D_H does not just constitute a first emotional reaction to the perception of stimuli, but that it can also partially explain the original relationships among TPB constructs. These results further confirm that A_D_H plays a crucial role in all aspects and stages of the intention formation process by influencing all the TPB constructs. Moreover, results show that attitude and Anticipated Happiness are the strongest predictors of intention in this context and that they both have a positive impact on it. Same reasoning for subjective norms, which are significant as well, but with a secondary relevance. Perceived behavioral control, instead, is the weakest predictor of the intention to buy the products and it has a significant path coefficient only in RQ5c, RQ4a, RQ4b, RQ4c.

**TABLE 10:** Path coefficients and indirect path coefficients.

	TPB Model	RQ3 Model	RQ4a Model	RQ4b Model	RQ4c Model	RQ5a Model	RQ5b Model	RQ5c Model
	Original TPB model	TPB with A_D_H as direct antecedent of I	TPB with A_D_H as direct antecedent of A	TPB with A_D_H as direct antecedent of SNs	TPB with A_D_H as direct antecedent of PBC	TPB with A_D_H as partial mediator of A → I	TPB with A_D_H as partial mediator of SNs → I	TPB with A_D_H as partial mediator of PBC → I
**Path coefficients**								
A → I	0.753***	0.544***	0.753***	0.753***	0.755***	0.547***	0.546***	0.549***
SNs → I	0.108***	0.083**	0.108***	0.108***	0.110***	0.084**	0.083**	0.085**
PBC → I	−0.058**	−0.047*	−0.058**	−0.058**	−0.066***	−0.047*	−0.047*	−0.055**
A_D_H → I		0.255***				0.250***	0.252***	0.251***
A_D_H → A			0.871***					
A_D_H → SNs				0.623***				
A_D_H → PBC					0.253***			
A → A_D_H						0.870***		
SNs → A_D_H							0.622***	
PBC → A_D_H								0.238***
**Indirect effect coefficients**								
A → I						0.217***		
SNs → I							0.157***	
PBC → I								0.060**
A_D_H → I			0.655***	0.067***	−0.017**			

***p < 0.01; **p < 0.05; *p < 0.1.

Potential collinearity among exogenous latent variables has been assessed using SPSS Statistic, through Variance Inflation Factors (VIF). To avoid multicollinearity problem, we considered as acceptable VIF values below or equal to 5 (Vercellis, 2009). Results show the absence of multicollinearity problems as all VIF values were lower than the above-mentioned threshold.

The model fit was assessed using a set of indicators, which include both relative and absolute indexes: χ^2^/degree of freedom (df), NFI, SRMR. The reason behind the use of χ^2^/df instead of χ^2^ relies on the fact that χ^2^ is not a reliable indicator when dealing with large samples (more than 200 observations), as it tends to suggest misfit, regardless of the true situation (Coughlan et al., 2008). χ^2^/df, instead, is more reliable with large samples. Hereafter, Table 11 presents the fit indexes for all the models: each model was built by incorporating each of the stated research questions in the original TPB framework. SRMR (Standardize Root Mean Square Residual) scores below 0.08 indicate an acceptable fit and NFI (Normed-Fit Index) is deemed to be acceptable when it is higher or equal to 0.95 (Coughlan et al., 2008). Even though there is no consensus about threshold values for χ^2^/df (Coughlan et al., 2008) we considered acceptable values those lower or equal to 3 (Iacobucci, 2010).

**TABLE 11:** R-square and Model fit indicators.

	TPB Model	RQ3 Model	RQ4a Model	RQ4b Model	RQ4c Model	RQ5a Model	RQ5b Model	RQ5c Model
	Original TPB model	TPB with A_D_H as a direct antecedent of I	TPB with A_D_H as a direct antecedent of A	TPB with A_D_H as a direct antecedent of SNs	TPB with A_D_H as a direct antecedent of PBC	TPB with A_D_H as a partial mediator of A → I	TPB with A_D_H as a partial mediator of SNs → I	TPB with A_D_H as a partial mediator of PBC → I
**R-Square**								
I	0.659	0.673	0.658	0.659	0.659	0.672	0.672	0.673
A			0.758					
SNs				0.389				
PBC					0.064			
A_D_H						0.756	0.387	0.057
**Model fit indicators**								
SRMR	0.072	0.066	0.077	0.077	0.072	0.067	0.148	0.269
χ^2^/df	2.374	3.030	3.084	3.068	3.037	3.029	3.955	4.613
NFI	0.834	0.841	0.838	0.839	0.840	0.841	0.792	0.757

Results indicate that RQ3, RQ4a, RQ4b, RQ4c, and RQ5a models perform slightly better in terms of model fit, compared to RQ5b and RQ5c models. Moreover, RQ3, RQ4a, RQ5a, RQ5b, and RQ5c models are aligned with previous studies that attempt to include emotional constructs within the TPB. Indeed, there is evidence that supports the inclusion of emotions as direct antecedents of intention (Londono et al., 2017), as direct antecedents of attitude (Ding et al., 2014; Chang, 2016) and as partial mediators of attitude, subjective norms and PBC on intention (Onwezen et al., 2014; Turel, 2016). RQ4b and RQ4c models, instead, enlarge previous findings by showing that Anticipated Happiness could be integrated into the TPB even as a direct antecedent of both SNs and PBC.

Since results confirmed a pervasive role of A_D_H within the TPB, we also assessed the goodness of two models in which A_D_H was simultaneously positioned as a predictor of A, SNs, PBC, and I and as partial mediator of A, SNs, and PBC on I. SEM analysis was then conducted on these two models. All path coefficients of the postulated relationships were significant, and each model was able to explain 67% of the variance in intention (*R*^2^_model a_ = 0.672; *R*^2^_model b_ = 0.673).

#### Structural Model by Product

We explored if there are any differences in the relationships between the constructs of the enlarged TPB (A, SNs, PBC, A_D_H, and I) produced by the type of product. The significance of the path coefficients of the inner model was slightly different when considering the two products separately. By modeling the enlarged TPB for the personal care product, SNs and PBC were found not to be relevant predictors of intention. An analogous result is achieved for the electronic device, where PBC did not represent a predictor of the intention to buy the product. Shifting the attention on the role of A_D_H in the enlarged TPB, all the proposed relationships are supported even considering the two products separately. This means that A_D_H was not strictly related to the product type, but it has a pervasive and strong role regardless of it.

### Discussion

All the postulated relationships between A_D_H and the TPB constructs were confirmed, suggesting the possibility to position A_D_H both as a direct antecedent of the four traditional TPB constructs and as a mediator of the relationships between intention’s predictors and intention itself. More in detail, A_D_H can be a direct predictor of attitude, meaning that A_D_H could be considered as a first signal that derives from an “emotional assessment” of the stimulus, feeding the subsequent creation of a positive attitude (Baumgartner et al., 2008). A_D_H also plays a role as a direct antecedent of SNs. A possible explanation for the underlying mechanism could be related to the projection bias (Loewenstein et al., 2003). Indeed, people who experience positive feelings about a product tend to think that their relevant others will also be pleased about it and consider purchase a good idea.

Moreover, A_D_H is also proved to be a predictor of PBC: this result can be better interpreted in light of the Construal Level Theory (CLT). CLT states that when Psychological Distance (PD) is high (in our case the focal product is new, the context trial is virtual and the purchase is not happening in the near future, thus PD can be considered high), people tend to focus on the desirability aspects of the behavior and to overlook more concrete ones such as the availability of resources and capabilities needed to use the product (Goodman and Malkoc, 2012). Thus, the desirability focus may trigger A_D_H which could lead the subject to perceive more control over the behavior, since feasibility considerations are not performed.

Finally, A_D_H is not just pervasive in the TPB, but it also has a tremendous impact on purchase intention, as is suggested by A_D_H’s ability to explain 0.587 of variance in intention when it is the only predictor. Thus, on the one hand, in the process of intention formation, A_D_H can be a first, immediate emotional response to the stimulus, impacting in turn on the more cognitive constructs of A, SNs, PBC, and I. On the other hand, A_D_H can, at least in part, explain the relationships between intention predictors and behavioral intention. Indirectly, the confirmation in a further study of the positive impact of A_D_H on TPB confirms the idea of A_D_E_s_ and A_Y_E_s_ as distinct constructs.

## General Discussion

This research shed some light on the ambiguous and fuzzy distinction between Anticipated and Anticipatory Emotions (Baumgartner et al., 2008). According to our results, Anticipatory Emotions are experienced in the present in relation to future events while Anticipated Emotions are forecasts of future emotional states, thus they occur when a person imagines a future situation and reasons about how he or she would feel in that context. Hence, Anticipatory Emotions reflect in physiological affective responses of individuals while Anticipated Emotions in the cognitive-emotional assessment. This outcome contributes to literature not only by providing further evidence to Baumgartner et al. (2008) theories, but also by offering a richer interpretation grounded onto objective autonomic measures confirming prior studies showing how physiological and self-reported measures tend to diverge as they tackle with different brain processes (e.g., Bettiga et al., 2017). The work thus proposes a strong methodological implication, suggesting a possible approach to use micro facial expressions analysis to study A_Y_E_s_.

We hence focused on the relationship between future-oriented emotions and intention to buy a new product, studying the relationships with the TPB’s intention predictors (Attitude, Subjective Norms, and Perceived Behavioral Control). Results suggested that Anticipated Happiness could qualify as a relevant antecedent of attitude (Anticipated Happiness was found to account for 89% of the variation in attitude), while Anticipatory Happiness seems to be more linked to perceptions of control (PBC), supporting the conceptualization of Anticipated Emotions as “cognitive-evaluative dimensions” and Anticipatory Emotions as “experiential-current reactions” (Carrera et al., 2012, p. 274). In particular, results show that higher levels of Anticipatory Emotions are expressed by subjects who declare lower levels of PBC. This suggests that either control may somehow inhibit the emotional reactions one has in terms of facial expressions, or that emotional reactions are overwhelming, so they trigger feelings of loss of control.

It seems reasonable that the cognitive construct of attitude, related to rational assessments of behavior, is mostly linked with Anticipated Happiness, which requires a cognitive effort of imagining future events and reasoning about one’s feelings in that hypothetical scenario. Similarly, since anticipatory emotional responses are experienced in the present (in line with Baumgartner et al., 2008), they are more overwhelming, and they are mostly related to a less cognitive construct, PBC, which is grounded on subjective perception than on rational evaluations. Indeed, the real control one has can be experienced only when the behavior is being performed, and it is better known as Actual Behavioral Control (Hassan et al., 2016).

Furthermore, this study enlarges previous findings in two main ways. First, previously tested relationships between Anticipated Emotions and the TPB variables have been verified and confirmed with a new construct, A_D_H, which belongs to the broader category of anticipated positive emotions, that have traditionally received less attention than anticipated negative emotions. A_D_H was found to be conceptually separated from attitude. Indeed, while attitude is more focused on one’s thoughts and evaluations toward a specific product, A_D_H captures the extent to which the product has a positive impact on the subject’s life, eliciting feelings of pleasure, and a sense of well-being. This result is in line with prior studies, showing that happiness and attitude are two distinct constructs with a positive effect of the first on the second one (Chang, 2016). Secondly, previous literature has been expanded by finding support for a new possible role of A_D_H as a direct antecedent of SNs and PBC. These results suggest that emotional responses may have an impact also on one’s perception of control over a behavior and over the perceived pressure to perform the behavior exerted by relevant others.

To conclude, Anticipated Happiness was found to have a pervasive role in all stages of the intention formation process, by qualifying at the same time as a direct antecedent of the traditional constructs and as a partial mediator of the existing relationships among the TPB constructs. These findings confirm the important role that happiness assumes in consumer choice (Mogilner et al., 2011) and the necessity to integrate emotional constructs in cognitive models, as the TPB, supporting prior studies moving in this direction (Kim et al., 2013; Onwezen et al., 2014; Turel, 2016; Londono et al., 2017). These results extend prior studies on anticipated emotions in purchasing process (Bagozzi et al., 2016) to a new context, highlighting the importance of considering emotional reactions generated in the encounter with a new product, to improve the capability to explain intention formation.

## Managerial Implications

Results of this study could have relevant implications for marketers involved in the design of advertisements and communication campaigns for new products. Indeed, the emotional identity of the viewer is likely to play a much prominent role than the rational one, thus becoming a strong predictor of the buying intention. Moreover, marketers are increasingly seeking to connect with customers on a more simple and essential level, by promising happiness and by creating brands that cultivate happiness. So, visual product communication should be designed in a way to elicit feelings of Anticipated and Anticipatory Positive Emotions (e.g., happiness) in the viewer. Of much less relevance are considerations about the opinions of important others or the perceived control over the behavior, thus these aspects should receive less attention. This task may not be as easy as it appears since happiness has been demonstrated to be a complex emotion who’s meaning often shifts among individuals.

Nevertheless, previous studies on the role of happiness in consumer research can offer interesting cues for eliciting it. For instance, it has been demonstrated that framing consumption as an experience instead of a mere purchase could create deeper connections with customers by evoking feelings of happiness (Nicolao et al., 2009; Bhattacharjee and Mogilner, 2014). Furthermore, the extent to which the experience is presented as “ordinary” or “extraordinary” has an impact on individuals’ happiness level and this effect is proved to be different between younger and older subjects (Bhattacharjee and Mogilner, 2014). Thus, age can be an important factor to take into consideration when presenting marketing content that frames consumption as an experience (Petersen et al., 2018).

Finally, the identification of two different categories of future-oriented emotions, namely anticipated and anticipatory ones, may support marketers in their communication strategies. Our results, indeed, show that anticipatory happiness is particularly relevant in situations of low perceived control while anticipated happiness is more related to a cognitive reasoning. This may provide directions to marketers on how to elicit happiness in high vs. low self-control consumers and which arguments could be more effective, especially when it comes to indulgent purchases. For instance, messages which provide customers with a reason to indulge (e.g., “luxury feels better earned”) resonate better with high self-control consumers, making them happier and more satisfied with the purchase. Conversely, for low self-control consumers, happiness is more likely to arise when the brand promotes spontaneous indulgence – e.g., “you don’t need a reason to indulge”- (Petersen et al., 2018). These findings have important implications for marketers which could increase the effectiveness of one-to-one communication, especially online, based on consumer personality variables, inferred from their online behavior data. For example, marketers can use social networks likes or language to infer personality traits of agreeableness and conscientiousness, which are correlated to the self-control trait.

## Limitations and Future Research

The study is subject to some limitations. The first limitation stems from the fact that, for time and resource constraints, the TPB model was just partially tested as the actual behavior of the participants was not measured. Although the behavioral intention is considered to be a good predictor of behavior (Ajzen, 1991), there is no guarantee that intention will translate into actual behaviors. Therefore, further research could address this issue by testing the relationship between actual behavior and the declared intention.

The second limitation regards the type of products used in the experiments: this research was limited to very new and innovative products presented through teaser videos. So, future research should replicate this study by using different kinds of products, to assess whether Anticipated and Anticipatory Happiness play the same role. This test could help in understanding whether Anticipated and Anticipatory Happiness are a function of the type of product and/or the purchase type. For instance, complex purchases could be characterized by a different decision-making process where emotional reactions could be offset by more rational and cognitive thoughts.

The third limitation is linked with the emotional construct under investigation. On the one hand, this study only investigates one positive emotion, happiness. Thus, further investigations could be aimed at assessing the role of other positive emotions like contentment, pleasure, and excitement, using the TPB model. On the other hand, the present study focuses only on Anticipated and Anticipatory Happiness. However, to further clarify the distinction between Anticipatory and Anticipated Emotions, other types of emotions (both positive and negative) should be considered.

The last limitation concerns the methods used to measure happiness. The construct was assessed using both self-reported and autonomic methods (micro facial expressions). However, it is reasonable to presume that A_Y_H does not only manifest through facial expressions, like changes in the lip corners and in the muscles near cheeks and eyes. Prior research (Kaklauskas et al., 2011) investigated other possible biometrical manifestations of this emotional construct, as the heart rate acceleration, the skin temperature, and blood pressure growth and the skin conductance change. So, results should be replicated with other types of autonomic measures.

## Data Availability Statement

The anonymized raw data supporting the conclusions of this article will be made available by the authors, without undue reservation, to any qualified researcher.

## Ethics Statement

Ethical review and approval was not required for the study on human participants in accordance with the local legislation and institutional requirements. The patients/participants provided their written informed consent to participate in this study.

## Author Contributions

Both authors listed have made a substantial, direct and intellectual contribution to the work, and approved it for publication.

## Conflict of Interest

The authors declare that the research was conducted in the absence of any commercial or financial relationships that could be construed as a potential conflict of interest.
